# Subgeneric division of the genus *Orcula* Held 1837 with remarks on Romanian orculid data (Gastropoda, Pulmonata, Orculidae)

**DOI:** 10.3897/zookeys.301.5304

**Published:** 2013-05-17

**Authors:** Barna Páll-Gergely, Tamás Deli, Atanas Irikov, Josef Harl

**Affiliations:** 1 Department of Biology, Shinshu University, Matsumoto 390-8621, Japan; 2 Békés Megyei Múzeumok Igazgatósága, Békéscsaba, Gyulai u 1. H-5600; 3 Department of Ecology and Environmental Conservation, Faculty of Biology, University of Plovdiv, 24 “Tzar Assen” Str., 4000 Plovdiv, Bulgaria; 4 Central Research Laboratories, Natural History Museum Vienna, Burgring 7, 1010 Vienna, Austria

**Keywords:** Anatomy, taxonomy, Alpine, Banatic, Illyric distribution

## Abstract

The genital anatomy of *Orcula jetschini* (Romania), *Orcula zilchi* (Bulgaria), and *Orcula wagneri* (Albania) is described. Based on anatomical features (morphology of the penial caecum) shell characters (sculpture and shape) and unpublished molecular data the genus *Orcula* is subdivided into three subgenera. *Orcula zilchi* was classified within the monotypic subgenus *Orcula (Hausdorfia)* **subgen. n.**; *Orcula jetschini*, *Orcula wagneri*, and *Orcula schmidtii* were classified to *Orcula (Illyriobanatica)* **subgen. n.** (type species: *Pupa schmidtii*) whereas the other *Orcula* species remain in the nominotypical subgenus. *Orcula (Hausdorfia)* is known from South-Eastern Bulgaria and North-Western Turkey, *Orcula (Illyriobanatica)* inhabits Western Romania, North-Western Greece, Albania, Macedonia, Kosovo, and Montenegro. The nine species of *Orcula (Orcula)* are known mainly from the Alps and the Western Carpathians (from Eastern France to Eastern Hungary and Slovakia).

The occurrence of only one *Orcula* species namely *Orcula jetschini* is verified from Romania. Available information suggests that data on the Romanian occurrence of *Orcula dolium* and *Orcula gularis* were based on wrongly identified specimens. *Sphyradium dobrogicum* (=*Orcula dobrogica*) is considered as a synonym of *Sphyradium doliolum*.

## Introduction

*Orcula* Held 1837, the type genus of the family Orculidae is a group of small (5–10 mm), pulmonate land snails with ovate–cylindrical shells and 3–4 lamellae within the aperture and the body whorl. Taxa are primarily found in mountainous regions in relatively humid habitats, most commonly in deciduous forests. There are at least 50 names described within the genus and 14 of them are considered as valid on species level (Harl et al. 2011). The Alps are inhabited by eight *Orcula* species, and this area is considered as the centre of the diversity of the genus. The type species, *Orcula dolium* (Draparnaud 1801), has the widest distribution within the genus, occurring from Eastern France to Eastern Slovakia and North-Eastern Hungary and several subspecies are recognized (Klemm 1967, Gittenberger 1978, Harl et al. 2011). Other Alpine *Orcula* species have much narrower areas and occur in the Alps of Austria, Italy and Slovenia only. Non-Alpine species include *Orcula jetschini* (Kimakowicz 1883) from Romania (Banat, Transylvania, Crişana), *Orcula schmidtii* (Küster 1843) from Montenegro, Albania and northwestern Greece, *Orcula wagneri* Sturany 1914 from Albania, Macedonia (FYROM) and Kosovo and *Orcula zilchi* Urbański 1960 is distributed from South-Western Bulgaria to North-Western Turkey.

The anatomy of the Alpine and the Illyric *Orcula* species is well-known (see Gittenberger 1978 and Schileyko 2012). On the other hand, the reproductive anatomy of the two Eastern European species (*jetschini* and *zilchi*) remained unpublished.

Gittenberger (1978) presented compelling data regarding the utility of the epiphallus and penial caecum (= ‘’Flagellum’’ or ‘’Penisflagellum’’) in taxonomic studies in *Orcula*. Gittenberger (1983) noted that extra-Alpine species had much stronger shell sculpture than Alpine species. These conchological and anatomical data, however, did not provide the resolution required to properly subdivide the genus.

Recently, Schileyko (2012) evaluated the taxonomic positions of most *Orcula* species. He concluded that the genus can be divided into two groups based on the morphology at the epiphallus–vas deferens transition. The transition is abrupt in *Orcula conica* (Rossmässler 1837), *Orcula fuchsi* Zimmermann 1931, *restituta* (Westerlund 1887), *spoliata* (Rossmässler 1837) and *Orcula dolium*, whereas it is gradual in *gularis* (Rossmässler 1837), *austriaca* Zimmermann 1932, *tolminensis* Wagner 1912, *wagneri* and *schmidtii*. It is not possible to assign some *Orcula dolium* specimens to one group or another. Independent of these two groups, Schileyko (2012) delineated five ‘’clusters’’ (species groups) on the basis of anatomical and conchological characters (1: *conica*, 2: *fuchsi*, 3: *dolium* (s. l.) + *spoliata*, 4: *austriaca* (s. l.) + *tolminensis*+ *gularis*, 5: *schmidtii*+ *wagneri*). In Schileyko’s phylogenetic scheme *Orcula conica* (unique shell shape and peculiar position of the penial caecum) and *Orcula fuchsi* (unique structure of the epiphallus) are the most basal members of the genus.

Recently, living specimens of *Orcula jetschini* and *Orcula zilchi* were made available for study. Anatomical investigation of these species allowed us to fully evaluate the taxonomic relationships within *Orcula*. We present data here that establishes subgenera within *Orcula* based on shell and genital characters. These divisions are further supported by unpublished molecular data (Harl et al. in prep.).

Furthermore, we discuss the orculid species reported from Romania. Two species, namely *Pupa (Orcula) jetschini* Kimakowicz 1883 and *Sphyradium dobrogicum* Grossu 1986 were originally described from Romania. Two other species (*Orcula dolium* and *Orcula gularis*) were also reported from Romania by Bielz (1863) and later by other authors. Distributions of the last two species (see Welter-Schultes 2012), unreliable data sources and inaccessible or lost voucher material make Romanian occurrence data questionable.

### Material and methods

The comprehensive map (Fig. 9) showing the distribution of *Orcula dolium*, Alpine endemic *Orcula* spp., *Orcula schmidtii*–*wagneri*, *Orcula jetschini* and *Orcula zilchi* were compiled by literature sources (Banak et al. 2012, Grossu 1986, Hausdorf 1996, Hlaváč 2002, Klemm 1973, Ložek 2006, Marković et al. 2004; Mitrović 2007, Mitrović and Jovanović 2000, Moine et al. 2005, Negrea 1962, 1966, Pintér and Suara 2004, Vavrova 2009, Páll-Gergely 2010, Stossich 1880, 1899), museum collections (HNHM, MMM, NHMW, SMF) and personal communications: O. Gargominy (France), W. de Mattia (Italy), P. Subai (Greece, Montenegro, France, Germany). Records of shells from deposits of the Tisza River (Pintér and Suara 2004) were excluded because of the unreliability of their origin.

Photographs of several focal planes were made with a Wild Makroskop M420 and a Nikon DS Camera Control Unit DS-L2. The different layers were combined with Helicon Focus 4.75 Pro to obtain one completely focused image.

Shells were directly observed without coating under a low vacuum SEM (Miniscope TM-1000, Hitachi High-Technologies, Tokyo). Teleoconch sculpture was noted on the dorsal or dorsolateral area of the penultimate whorl.

### Abbreviations

**HNHM** Magyar Természettudományi Múzeum (Budapest, Hungary)

**MMM** Munkácsy Mihály Múzeum (Békéscsaba, Hungary)

**MNINGA** Muzeul Național de Istorie Naturală “Grigore Antipa” (Bucharest, Romania)

**NHMSB** Natural History Museum, Sibiu (Romania), Bielz collection

**NHMSK** Natural History Museum, Sibiu (Romania), Kimakowicz collection

**NHMW** Naturhistorisches Museum Wien (Vienna, Austria)

**SMF** Senckenberg Forschungsinstitut und Naturmuseum (Frankfurt am Main, Germany).

**SP** Collection Péter Subai (Aachen, Germany)

## Results

### Systematics Family Orculidae Pilsbry 1918

#### Genus Orcula

Held 1837

http://species-id.net/wiki/Orcula

Orcula Held, Isis: 919. (1837)

##### Type species.

*Pupa dolium* Draparnaud 1801, by subsequent designation Gray: 1847: 176.

##### Diagnosis.

Shell yellowish–greenish to dark brown; cylindrical to conical and elongated; 8–10 weakly convex whorls; sculpture of first 0.5–1.0 protoconch whorl usually smooth, but may be of fine spiral lines, which may be extremely weak; teleoconch axial sculpture variable, ranging from irregular growth lines to equally spaced, conspicuous radial structure; apertural barriers: one parietal and 1–3 columellar lamellae; palatal side of the aperture smooth or with strong tooth or thickening parallel to the apertural lip; parietal callus weak, subangularis sometimes present; palatalis plicae missing.

Penis cylindrical, penial caecum of variable length and shape; penial appendix absent; interior of penis, epiphallus and caecum with longitudinal folds; retractor muscle attaches to the penis-epiphallus junction on the opposite side of the penial caecum; diverticulum absent; distal part of vas deferens sometimes slightly swollen, entering epiphallus terminally; bursa copulatrix long, club-like.

##### Habitat.

*Orcula* species occur in humid limestone areas, usually forests, or rocky boulder fields at high altitudes. Animals live under stones, leaf litter or decaying wood, or at the base of large rocks.

##### Remarks.

Detailed anatomical and conchological diagnoses were provided by Gittenberger (1978) and Schileyko (1998). According to Hausdorf (1996), the genera *Orcula*, *Orculella* Steenberg 1925 and *Schileykula* Gittenberger 1983 cannot be distinguished from each other based on conchological characters alone.

Some African genera, such as *Fauxulus* Schaufuss, *Fauxulella* Pilsbry and *Anisoloma* Ancey have very similar genital tracts but usually possess sinistral shells with several apertural lamellae and denticles (see Schileyko 1998, 2012).

In general, species of *Schileykula* and *Orculella* usually inhabit dry limestone areas in the Mediterranean. The only exceptions known are the closely related *Orculella bulgarica* (Hesse) and *Orculella aragonica* (Westerlund) which both prefer very humid, marshy stream banks (Garrido et al. 2005, Arrébola et al. 2012).

**Figure 1.**
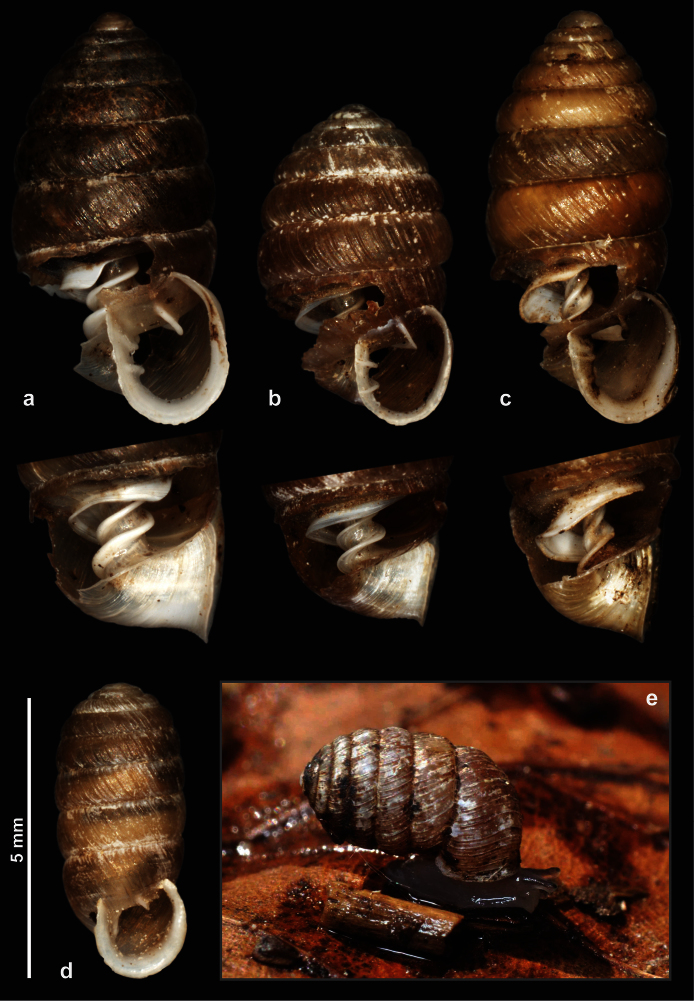
Shells and a living specimens of Orculidae species. **a** *Orcula (Orcula) dolium* (Draparnaud 1801), Hungary, Bükk Mts., Farkasnyak, Vöröskő, leg. Németh, L., 21.07.1984 **b** *Orcula (Illyriobanatica) jetschini* (Kimakowicz 1883), Romania, Jud. Bihor, Munţii Pădurea Craiului, Şuncuiuş, Valley of Crişul Repede, in front of Peştera Vantului (cave), limestone, leg. Bata, Danyik, Deli, 11.04.2011. **c** *Orcula (Hausdorfia) zilchi* Urbański 1960, Turkey, Vil. Bursa, between Bozüyük and İnegöl, by the “Mezit 7” bridge, limestone rocks and beach forest next to the road, 580 m, 39°55.724'N, 29°43.939'E, leg. Páll-Gergely, B., 30.09.2007 **d** *Sphyradium doliolum* (Bruguière 1792), Romania, Jud. Tulcea, Forest near the Cocoş Monastery, 145 m, 45°12.835'N, 28°24.415'E. Leg: Németh, L. & Páll-Gergely, B. 26.05.2011 **e** same locality as b. Photos: J. Harl (**a**–**d**) and T. Deli (**e**).

**Figure 2.**
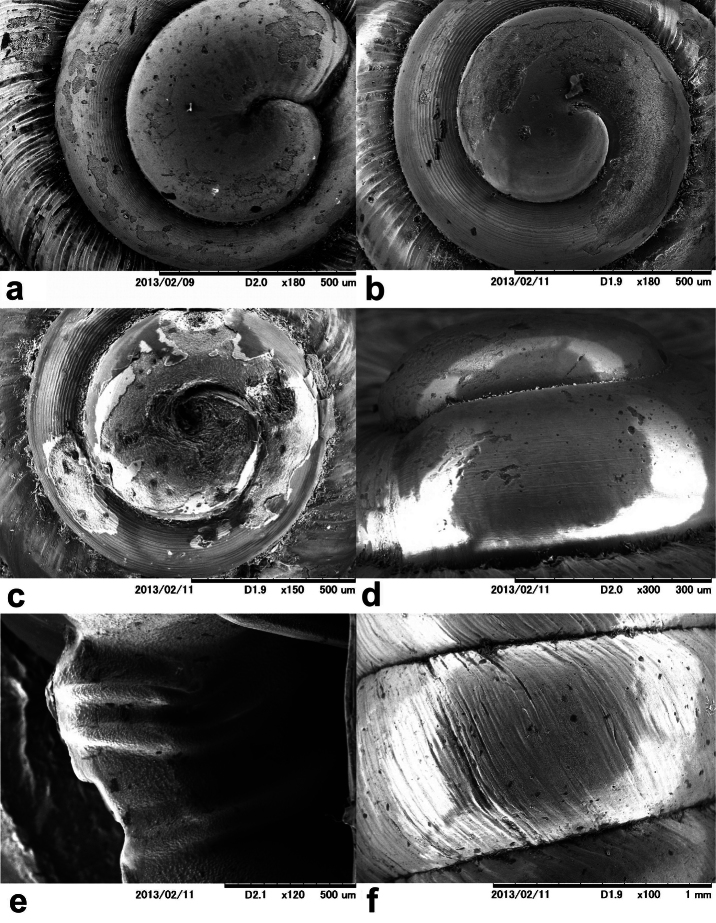
SEM of shells of various *Orcula* taxa. **a** protoconch of *Orcula (Orcula) dolium* (Draparnaud, 1801), Hungary, Bükk Mts., Farkasnyak, Vöröskő, leg. Németh, L., 21.07.1984 **b** protoconch of *Orcula (Illyriobanatica) schmidtii* (Küster 1843), Albania,Mirditë Mts., 1 km NE of Ndërshenë, beneath the Gurit te Çikut peak, 1350 m, 41°49.952'N, 20°06.034'E, leg. Erőss, Fehér, Kontschán, Murányi, 21.10.2002 **c** protoconch of *Orcula (Hausdorfia) zilchi* Urbański 1960, Turkey, Vil. Bolu, Abant Gölü N, 1030 m, 40°38.756'N, 31°21.531'E, leg: Páll-Gergely, B., 17.05.2006 **d** protoconch of *Orcula (Orcula) austriaca* Zimmermann 1932, Austria: Niederösterreich, Piestingtal, Waldegg, 412 m, 47°52.293'N, 16°2.722'E, Duda, M., Haring, E., Harl, J., Kruckenhauser, L., Sattman, H, 10.09.2009 **e** columellar lamellae of *Orcula (Hausdorfia) zilchi*, locality: see figure **2c f** sculpture of *Orcula (Orcula) tolminensis* Wagner 1912, Austria, Kärnten, Karawanken, Eisenkappel, Kupitzklamm, 674 m, 46°27.979'N, 14°36.915'E, leg. Duda, M., Haring, E., Harl, J., Kruckenhauser, L., Sattman, H., 29.07.2009.

**Figure 3.**
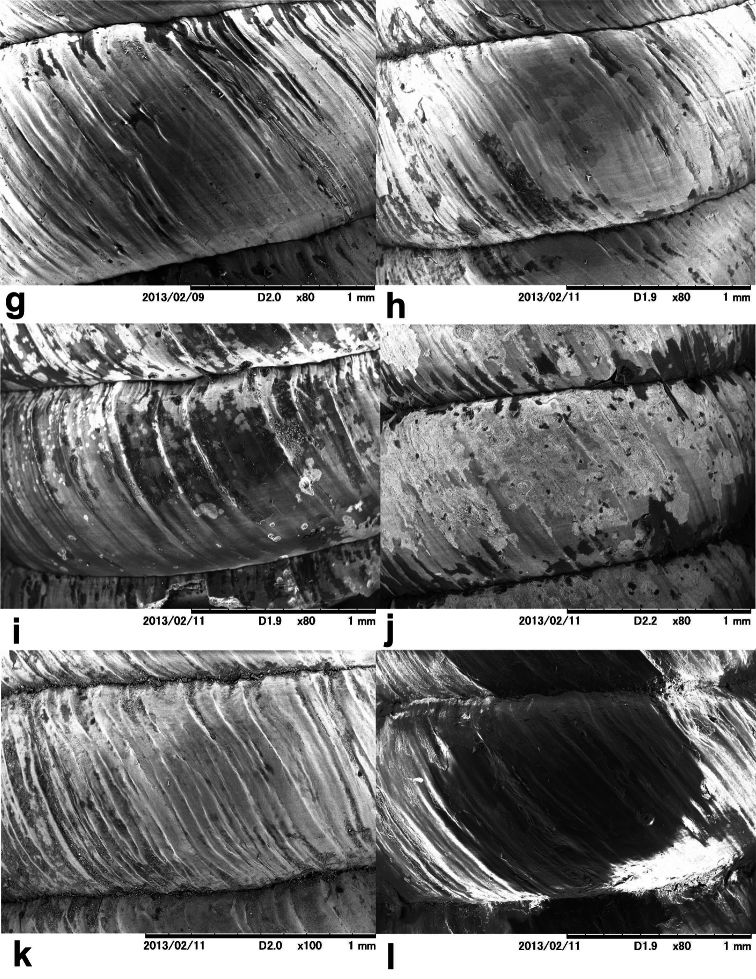
SEM of the shell sculpture of various *Orcula* taxa. G *Orcula (Orcula) dolium* **H** *Orcula (Orcula) austriaca* **I** *Orcula (Illyriobanatica) jetschini*, Romania, Jud. Bihor, Munţii Pădurea Craiului, Şuncuiuş, Valley of Crişul Repede, in front of Peştera Vantului (cave), limestone, leg. Bata, Danyik, Deli, 11.04.2011. **J** *Orcula (Illyriobanatica) wagneri* Sturany 1914, Albania, Malësia e Madhe, 11 km from Bogë, north of Tërthorës pass, 1800 m, 42°23.537'N, 19°43.782'E, leg. Erőss, Fehér, Kontschán, Murányi, 20.10.2002. **K** *Orcula (Illyriobanatica) schmidtii*, locality: see figure **2b L** *Orcula (Hausdorfia) zilchi*, locality: see figure **2c**.

**Figure 4.**
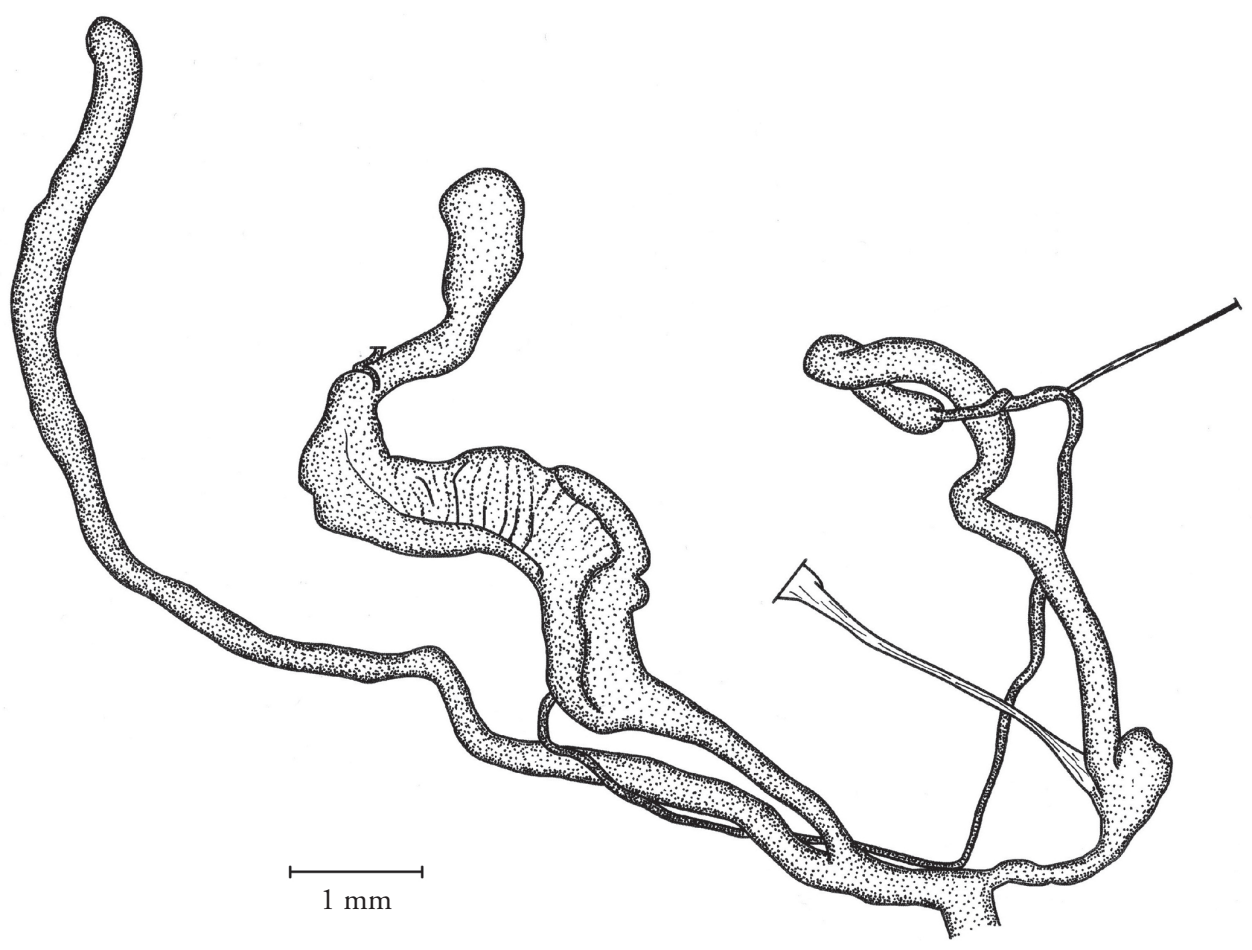
Genital anatomy of *Orcula (Illyriobanatica) jetschini* (Kimakowicz 1883), locality: see figure **1b**.

**Figure 5.**
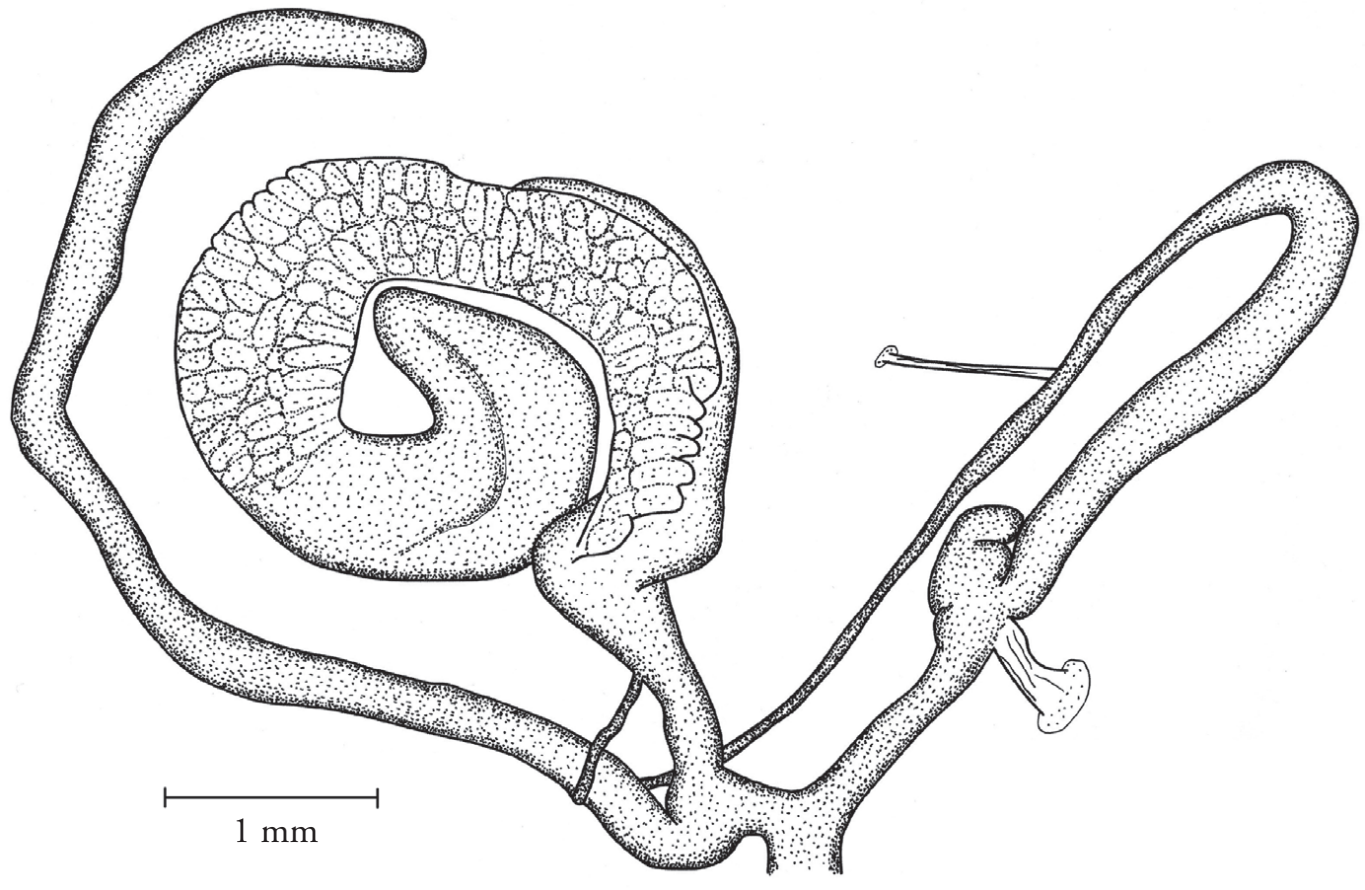
Genital anatomy of *Orcula (Illyriobanatica) wagneri*. Albania, Bjeshkët e Nemuna (Prokletije Mts), above village Okol, near pass Qafa e Pejës, W slope of Mt. Maja e Popluks, at a spring on limestone, 1660 m, 42°27.343'N, 19°46.478'E, leg. Barina Z, Puskás G, Sárospataki B, 16.07.2010.

**Figure 6.**
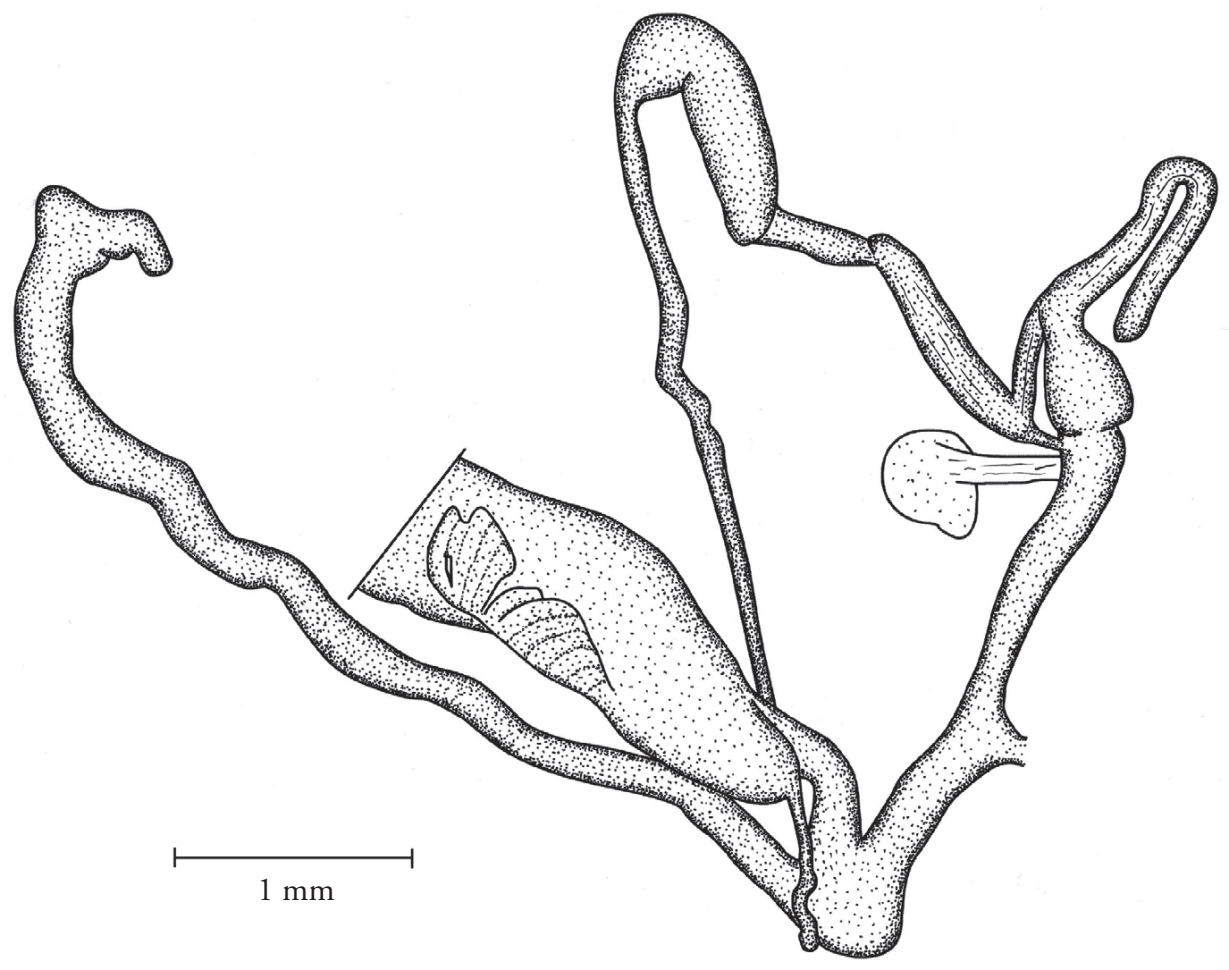
Genital anatomy of *Orcula (Hausdorfia) zilchi* Urbański 1960. Bulgaria, Strandzha Mts., Kondolovo village, 42°6.150'N, 27°39.896'E, leg. Irikov A, 28.04.2012.

**Figure 7.**
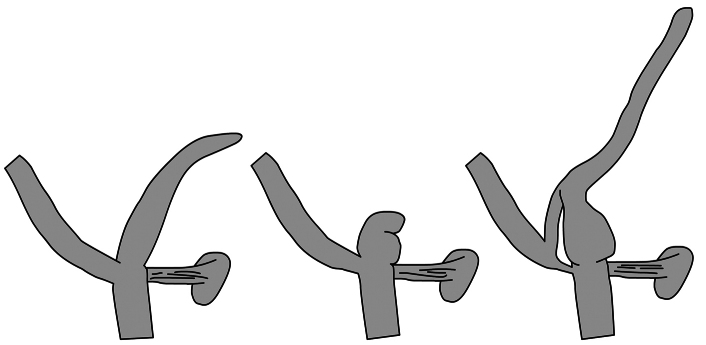
Schematic drawings of the penial caecum of *Orcula* subgenera. left: *Orcula (Orcula)*, middle: *Orcula (Illyriobanatica)*, right: *Orcula (Hausdorfia)*.

**Figure 8.**
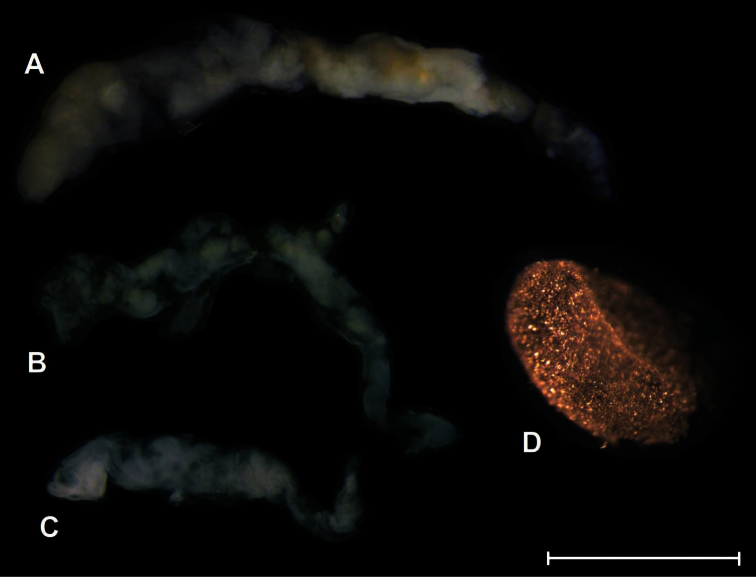
Spermatophores. **A** *Orcula (Illyriobanatica) jetschini* (Kimakowicz 1883) **B** *Orcula (Illyriobanatica) wagneri* and **C** *Orcula (Hausdorfia) zilchi* **D** egg. *Orcula (Hausdorfia) zilchi*; spermatophore and egg from different individuals. Scale = 1 mm.

**Figure 9.**
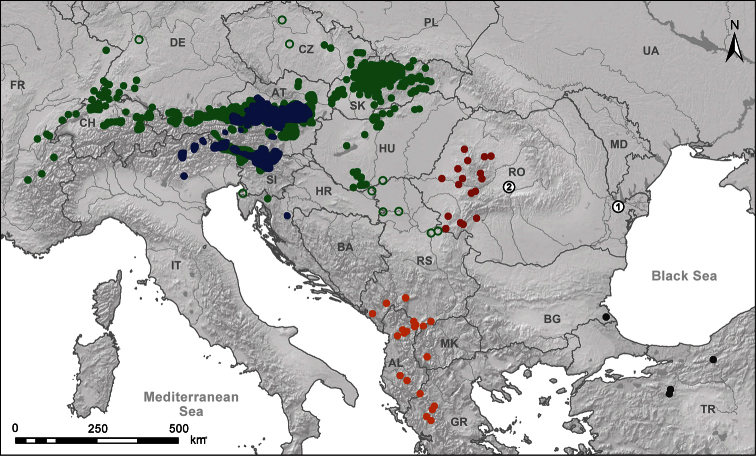
Distribution map of *Orcula*. Subgenus *Orcula*: *Orcula (Orcula) dolium* (green) (green circles indicate fossil records), Alpine endemic species (blue); subgenus *Illyriobanatica*: *Orcula (Illyriobanatica) wagneri* and *Orcula (Illyriobanatica) schmidtii* (orange), *Orcula (Illyriobanatica) jetschini* (red); subgenus *Hausdorfia*: *Orcula (Hausdorfia) zilchi* (black). Number **1**: type locality of *Sphyradium dobrogicum* Grossu 1986; Number **2** locality of *Orcula gularis* (Rossmässler 1837) according to Bielz (1863).

#### Subgenus Orcula

##### Diagnosis.

Shell smoothish with irregular growth lines; apex somewhat conical, not blunt; aperture with 2–3 columellar lamellae; penial caecum simple and usually longer than half the length of the penis; its base often not conspicuously thickened.

##### Content.

*austriaca*, *conica*, *dolium*, *fuchsi*, *gularis*, *pseudodolium* Wagner 1912, *restituta*, *spoliata*, *tolminensis*.

##### Remarks.

The soft anatomy of various *Orcula* taxa has been described in the following papers: *austriaca* (Gittenberger 1978, Schileyko 2012), *austriaca faueri* Klemm 1967 (Gittenberger 1978, Schileyko 2012), *austriaca pseudofuchsi* Klemm 1967 (Gittenberger 1978, Schileyko 2012), *conica* (Soós 1925, Gittenberger 1978, Schileyko 2012), *dolium* (Soós 1917, 1925, Steenberg 1925, Gittenberger 1978, Varga 1986, Grossu 1987, Reischütz 1995, Schileyko 1984, 1998, 2012), *dolium brancsikii* Clessin 1887 (Reischütz 1995), *dolium edita* Ehrmann, 1933 (Schileyko 2012), *dolium gracilior* Zimmermann 1932 (Gittenberger 1978, Schileyko 2012) *dolium infima* Ehrmann, 1933 (Schileyko 2012), *dolium pseudogularis* A. J. Wagner, 1912 (Gittenberger 1978), *fuchsi* (Gittenberger 1978, Schileyko 2012), *gularis* (Soós 1925; republished by Grossu 1987, Gittenberger 1978, Schileyko 2012), *pseudodolium* (Gittenberger 1978), *restituta* (Gittenberger 1978), *spoliata* (Gittenberger 1978, Schileyko 2012), *tolminensis* (Gittenberger 1978).

The penial caecum of *Orcula (Orcula) restituta* is very short compared to other *Orcula (Orcula)* species, but the shell is similar to that of *Orcula (Orcula) gularis*. Prior to Klemm (1967), *restituta* was considered a subspecies of *gularis*.

A third columellar lamella is rarely present, but can occur in a small percentage of individuals within a population. Brancsik (1888: 84) noted a third columellar fold in only one individual of thousands in each *Orcula dolium titan* (Brancsik 1888) and *Orcula dolium dolium*.

##### Distribution.

Most species have limited distributions in the Alps (mainly Austria). *Orcula dolium* is widely distributed in Central Europe, in the Alps (eastern France, Switzerland, Southern Germany, Northern Italy, Austria, Slovenia, Northern Croatia, and Slovenia) and the Western Carpathians (Northern Hungary, Slovakia, Eastern Czech Republic). The Croatian records of *Orcula dolium* and *Orcula gularis* (Stossich 1880, 1899, Zimmermann, 1932) have not been verified by recent investigations.

Our knowledge of the distribution of *Orcula dolium* is distorted due to misidentified material. Probably all reports of this species (living and fossil) from Spain (e.g. Llamas et al. 1995) refer to *Orculella aragonica* (see Arrébola et al. 2012). Italian (Toscana) records (Zanchetta et al. 2004, 2006) refer to a yet unknown *Orculella* species (see photo in Zanchetta et al. 2006). Damjanov and Likharev (1975) reported *Orcula dolium* from the Balkan Peninsula, South, Central and West Europe, the Crimea, Western Ukraine, Central Asia, Tunisia, Ethiopia and northen Iran. This distribution is much broader than that of *Orcula dolium* and probably refers to the distribution of the family Orculidae. Likharev and Rammelmejer (1952) and Sysoev and Schileyko (2009) speculated that *Orcula dolium* occurs in Ukraine. This supposition has been included in distribution maps (Welter-Schultes, 2012), but to date the taxon’s occurrence in Ukraine has not been verified data (Balashov and Gural-Sverlova 2012). Soós (1943) mentioned that during careful collections around Munkács (Mukachevo, southwest Ukraine), Traxler was not able to find the species.

*Orcula dolium* was more widely distributed during the Pleistocene. The northernmost localities were published by Ložek (2006) (Czech Republic, ca, 30 km north of Prague) and Moine et al. (2005) (Germany, northern Baden-Württemberg). The southernmost locality was reported by Mitrović (2007) from the Serbian Kisiljevo.

Sacco (1897) described *Orcula dolium* var. *pliopedemontana* from the middle Pliocene sediments at Ceresole d’Alba (Italy: ‘’Villafranchiano’’). The description is unfortunately insufficient and the taxonomic position of this form is uncertain (Ferrero-Mortara et al. 1984, Pilsbry 1922). More recently, Ciangherotti et al. (2007) made no mention of the species from the same sediment layers.

#### Orcula (Illyriobanatica)

Páll-Gergely & Deli subgen. n.

##### Type species.

*Pupa (Orcula) jetschini* M. von Kimakowicz 1883.

##### Diagnosis.

Shell usually with strong axial sculpture (irregular ribs), with two columellar lamellae, apex rather rounded, not attenuate. The penial caecum usually consists of two parts (‘’tubercles’’) and its length is less than half that of the penis.

##### Etymology.

The name of this new subgenus refers to its distribution in the Illyrian and Banatic biogeographical regions. It is feminine.

##### Content.

*jetschini*, *schmidtii* and *wagneri*.

##### Distribution.

Montenegro, Albania, Northwestern Greece, Kosovo, Macedonia (*Orcula wagneri* and *Orcula schmidtii*) and the western part of Romania (*Orcula jetschini*).

##### Remarks.

The reproductive anatomy of *Orcula schmidtii transversalis* (Westerlund 1894) was described by Hausdorf (1987) and Reischütz and Sattmann (1990). According to Hausdorf (1987), the penial caecum of *Orcula schmidtii transversalis* is short, but simple. The caecum appears double in the illustration provided by Reischütz and Sattmann (1990).

#### Orcula (Illyriobanatica) jetschini

(Kimakowicz 1883)

http://species-id.net/wiki/Orcula_jetschini

Pupa (Pupilla) dolium , Bielz, Fauna der Land- und Süsswasser-Mollusken Siebenbürgens, 89. (1863)
Pupilla dolium , — Bielz, Fauna der Land- und Süsswasser-Mollusken Siebenbürgens: 94–95. (1867)
Pupa (Orcula) jetschini Kimakowicz, M. von. — Verhandlungen und Mittheilungen, 33: 44–46. (1883)
Orcula jetschini , — Pilsbry: Manual of Conchology: 5, 17., Plate 2, fig. 10–11 . [‘’Transylvania, restricted to the southwestern part: Vajda-Hunyad and Bad Gyogy (Kimakowicz), Judenberg near Zalatna (Jickeli). Cerna valley at Mehadia in the Banat (Jetschin) ’’]. (1922)
Orcula jetschini , — Soós: A Kárpát-medence Mollusca faunája: 155–156, plate 6, fig. 18. (1943)
Orcula jetschini , — Kerney et al. Die Landschnecken Nord- und Mitteleuropas: 102–103. (1983)
Orcula (Orcula) dolium (partim: citation of Bielz 1863), — Grossu, Gastropoda Romaniae 2: 221–223. (1987)
Orcula (Orcula) jetschini , — Grossu, Gastropoda Romaniae 2.: 223–224. (1987)
Orcula jetschini , — Harl et al. Archiv für Molluskenkunde, 140 (2): 184, Plate 4, fig. J. (2011)
Orcula jetschini , — Welter-Schultes, European non-marine molluscs: 145. (2012)

##### Material.

RO, Jud. Bihor, Munţii Pădurea Craiului, Şuncuiuş, Valley of Crişul Repede, in front of Peştera Vantului (cave), limestone, leg.: Bata, Danyik, Deli, 11.04.2011. (anatomically examined); RO, Gyalui-havasok (Munţii Gilăului), Runki szakadék (gulch of Runk), leg. Papp, J., 22.07.1959, HNHM 73030/3; RO, Bihar Mts (Munţii Apuseni)., Felsőgirda (Gârda de Sus), Ordincus valley., leg. Kovács, Gy., 30.05.1985, HNHM 68284/2; RO, Muntii Bihorului, Baita, Piatra Graitoare, environment of the Crisu Baitei River, leg. Kovács, Gy., 23.08.1974, HNHM 68283/2; Černath. (Chernathal) bls Badern (?) v. Mehadia, leg. Jetschin 1882, NHMSK 4874/5; Forstgra (Forstgartens) bei im Černathal, Banat, leg. Jetschin 1882, NHMSK 4875/2; Klausenburg, Györgyfalvaer Wald, leg. Marzlof 1891, NHMSK 7470/8; Zalathna gegen den Judenberg, leg. Barth 1866–1906, NHMSK 7468/8; Banat, Herkulesbad, leg. Deubel 1895 May–Juni, NHMSK 7469/6; Steierdorf bis zur Höhle Panur, leg. Jetschin 1885, NHMSK 4876/4; Černathal b. Mehadia, leg. Jetschin 1885, NHMSK 4877/4 (‘’*mut. albina*’’); Gyógybad nächst Broos. Orm. (?) 1887, NHMSK 4873/3; Hideg-Szamos, NHMSB 51/72, 50136–50137; Györgyfalvaer Wald b. Klausenburg, NHMSB 51/82, 50389–50391; Unter-Grohob bei Körösbánya NHMSB 51/92, 50713; Klausenburg, Bükk, NHMSB 51/13, 48225–48226; Klausenburg ?? (not legible on label) Wald, NHMSB 51/23, 48500; RO, Bihor Mts., Valea Boghii (valley), 46°36.610'N, 22°39.542'E, leg. Páll-Gergely, B. 09.08.2007.; RO, Jud. Bihor, Bălnaca Groşi, cliffs at Bíró Lajos cave., leg. Domokos, T. 18.04.2004, MMM 04503/1; Munţii Pădurea Craiului, Şuncuius valley of Crişul-Repede, under shrubs., leg. Domokos, T. & Deli, T., 08.07.2005, MMM 04505/2; Munţii Pădurea Craiului, Şuncuiuş valley of Mişid-brook near brook-Alnetum., leg. Domokos, T.& Deli, T., 08.07.2005, MMM 04506/3; Munţii Apuseni, Gârda de Sus, Ordincus valley., leg. Domokos, T. & Kovács, Gy., 30.05.1985, MMM 04501/2; Munţii Apuseni, Gârda de Sus, Ordincus valley., leg. Domokos, T. & Deli, T., 30.11.2009, MMM 92488/1; Munţii Zărandului, Troaş, Pietroasia, floating debris., leg. Domokos, T., 07.06.2002, MMM 04502/10; Munţii Zărandului, Troaş, Valea Galsa floating debris, leg. Domokos, T. et al., 27.05.2005, MMM 04504/11; Jud. Arad, above Obârşia (Munţii Metaliferi) (1.9 km W of Arad-Hunedoara board), forest clearing (*Corylus*), 700 m, leg. Deli, T. & Domokos, T, 07.03.2007, MMM 90866/2; Jud. Arad, between Pojoga and Căprioara (7 km SE Săvârsin), in gorge–forest, 120 m, leg: Deli, T., Domokos, T., Páll-Gergely, B., Subai, P., 15.04.2007, MMM 91092/2; Jud. Arad, between Pojoga and Căprioara (7 km SE Săvârsin), in gorge–forest, 120 m., leg: Deli, T., 12.06.2007, MMM 90814/1; Jud. Caraş-Severin, between Moldova Nouă and Padina Mate, forest (*Fagus*, *Caprinus*, *Ruscus*) with limestone rocks, 300 m, leg. Boldog, G., Deli, T., Kóra, J., 04.07.2007, MMM 92489/1; Mehadia Mts. (Munţii Mehedinţi), Cerna valley, Jelărăului gorge, above Băile Herculane, large flotsam deposit, leg. Boldog, G., Deli, T., Kóra, J., 08.07.2007, MMM 92494/6; Munţii Mehedinţi, Cerna valley, Jelărăului gorge, above Băile Herculane, large flotsam deposit, leg. Deli T., Horváth, É., Lennert, J., Páll-Gergely, B., Subai, P., 04.05.2008, MMM 92490/5; Munţii Vâlcan, N Tismana, near Monastery Tismana, bank of Tismana brook, flotsam deposit., leg. Deli, T., Domokos, T., Páll-Gergely, B., Subai, P., 15.04.2007, MMM 92491/1; Munţii Vâlcan, Piscuri-valley, 1,4km N Vâlcele (NE Tismana), flotsam deposit., leg. Deli T., Domokos T., Páll-Gergely B., Subai, P., 17.04.2007, MMM 92492/1; Munţii Vâlcan, N of Runcu, Cheile Sohodol, 4.5 km upstream of the gorge entrance, limestone walls., leg. Boldog, G., Deli, T., Kóra, J., 06.07.2007, MMM 92493/1.

##### Description of the genitalia.

Two specimens were anatomically examined. Penis slim, with the retractor muscle attached at its distal end; penial caecum very small, vestigial, consisting of two “tubercles”; epiphallus very long and cylindrical; there is clear distinction between the vas deferens and the epiphallus; vas deferens long and relatively thick; a slender retractor muscle is attached near the proximal end. Vagina short and thick, but pedunculus relatively long; bursa copulatrix extremely long, with the distal end slightly expanded. In one specimen an elongated, simple spermatophore was found with the apical portion slightly thickened.

##### Distribution.

*Orcula (Illyriobanatica) jetschini* is known only from western part of Romania (Banat, Crişana and Western Transylvania). The Hungarian record (Pintér and Suara 2004) is apparently based on a flotsam specimen so its origin is suspect (Varga, 2009). Negrea (1966) reported the species from Moldova Nouă, which lies close the Serbian border and, therefore, it is expected to occur in Serbia. The Pleistocene distribution of *Orcula dolium* included the ‘’Požarevac Danube Area’’ (Mitrović 2007), which is just on the other bank of the Danube River, but temporal and spatial sympatry of *Orcula (Illyriobanatica) jetschini* and *Orcula (Orcula) dolium* is not verified.

##### Ecology.

The species inhabits deciduous forests. It is found most commonly between small stones and leaf litter on the forest floor or under hazelnut (*Corylus*) bushes. The species is known from non-limestone bedrock, such as the Zarand Mountains.

##### Conservation status.

Least concern (LC) according to IUCN criteria (Fehér 2011).

##### Remarks.

All living specimens found were covered in mud, causing them to appear like tiny grains of soil. The ribbed shell is possibly an adaptation for camouflaging. The photographs herein are of cleaned shells.

#### Orcula (Illyriobanatica) wagneri

Sturany 1914

http://species-id.net/wiki/Orcula_wagneri

Orcula wagneri Sturany in Sturany and Wagner, — Denkschriften der Kaiserlichen Akademie der Wissenschaften, mathematisch-naturwissenschaftliche Klasse 91: 45, Plate 15, fig. 82b. (1914)
Orcula wagneri , — Harl et al., Archiv für Molluskenkunde, 140 (2): 186, Plate 5, fig. A–G, J. (2011)
Orcula wagneri , — Audibert, Folia Conchyliologica 14: 21–25. Figure 1: habitat, figure 2, and Figure 1, 2, 4, 5: shells with possible signs of parasites. (2011)
Orcula wagneri , — Schileyko, Ruthenica, 22 (2): 152-253, 156, figure 17 (genitalia). (2012)
Orcula wagneri , — Welter-Schultes, European non-marine molluscs:146. (2012)

##### Material.

Albania, Bjeshkët e Nemuna (Prokletije Mts), above village Okol, near pass Qafa e Pejës, W slope of Mt. Maja e Popluks, at a spring on limestone, 1660 m, 42°27.343'N, 19°46.478'E, leg. Barina, Z., Puskás, G., Sárospataki, B., 16.07.2010., HNHM 98841.

##### Description of the genitalia.

One specimen was dissected. Penis cylindrical and slim, with a short, but thick penial caecum, the proximal portion broader than the short and slimmer distal portion; retractor muscle attaches at the penis–epiphallus transition; epiphallus more than twice as long as the penis and much thicker, its transition to the vas deferens is gradual, barely discernable; there is a slim retractor muscle attached to the proximal portion of the epiphallus; proxim\al portion of the vas deferens thicker than the distal part. Vagina and free pedunculus extremely short; bursa copulatrix almost twice as long as the combined length of the penis–epiphallus complex.

##### Conservation status.

*Orcula (Orcula) wagneri* is listed as Near Threatened (NT), being close to the criteria threshold for Vulnerable (Pall-Gergely 2011a).

##### Remarks.

Our observations on the genitalia agree with that of Schileyko (2012), who investigated the anatomy of *Orcula wagneri* from the Tomor Mountains (‘’Maja e Tomorit Mt., S Albania’’). A partially reabsorbed, elongated, spermatophore was located in the bursa copulatrix (Fig. 8B).

#### Orcula (Hausdorfia)

Páll-Gergely & Irikov subgen. n.

##### Type species.

*Orcula zilchi* Urbański 1960 (by monotypy).

##### Diagnosis.

Shell with conical apex and strong axial sculpture (irregular axial growth lines), with three columellar lamellae (columellar, supracolumellar and one short lamellae above), palatalis reaches its maximum height on the dorsolateral side. Penial caecum very long with thickened base, canal connecting the proximal end of the epiphallus to the penial caecum.

##### Etymology.

The new subgenus is named in honour of Dr Bernhard Hausdorf (University of Hamburg), who first noted the unusual shell characters of *Orcula zilchi* and questioned its generic status (Hausdorf 1996). It is feminine.

##### Distribution.

See under *Orcula (Hausdorfia) zilchi*.

##### Remarks.

According to Schileyko (2012), the penial caecum of *Orcula fuchsi* is long and slender, with a thickened base, making it similar in morphology to *Orcula zilchi*. However, the characteristic canal connecting the proximal end of the epiphallus with the penial caecum of *Orcula zilchi* is lacking in *Orcula fuchsi*. The long caecum of this species is also illustrated by Gittenberger (1978), but its base is not conspicuously thickened. This may vary between populations or during an individual’s life history.

#### Orcula (Hausdorfia) zilchi

Urbański 1960

http://species-id.net/wiki/Orcula_zilchi

Orcula zilchi Urbański, J., Bulletin de la Société des Amis des Sciences et des Lettres de Poznan (Série D) 1: 57. [‘’Am rechten Ufer des Ropotamo, etwa 3 km vor seiner Mündung (etwa 30 km südlich von Burgas) ’’]. (1960)
Orcula zilchi , — Damjanov and Likharev, Fauna Bulgarica, Gastropoda terrestria, vol. IV: 115. (1975)
Orcula (?) *zilchi*, — Hausdorf, Archiv für Molluskenkunde125 (1/2): 14, Plate 1, fig. 1. [‘’Westanatolien: V. Kütahya, Safa 2 km R Domaniç’’]. (1996)
Orcula zilchi , — Páll-Gergely, Zoology in the Middle East, 50: 91. (2010)
Orcula zilchi , Harl et al. — Archiv für Molluskenkunde, 140 (2): 186–187, Plate 4, fig. F, G. (2011)
Orcula zilchi , — Welter-Schultes, European non-marine molluscs:146. (2012)

##### Material.

Bulgaria, Strandzha Mts., Kondolovo village, 42°6.150'N, 27°39.896'E, leg. Irikov, A., 28.04.2012. (anatomically examined); Bulgaria, Silkosiya Reserve, near Kosti Village, 23.06.2001, leg., А. Irikov; Turkey, Vil. Bolu, Abant Gölü N, 1030 m, 40°38.756'N, 31°21.531'E, leg. Páll-Gergely, B., 17.05.2006.; Turkey, Vil. Bursa, between Bozüyük and İnegöl, by the ‘’Mezit 7’’ bridge, limestone rocks and beach forest next to the road, 580 m, 39°55.724'N, 29°43.939'E, leg. Páll-Gergely, B., 30.09.2007; Bulgaria, floating debris 6 km N of Malko Tarnovo, 210 m, UTM NG 45, 42°5.028'N, 27°25.698'E, leg. Dedov & Subai 8.5.2008, SP 22168/2 (juv.)

##### Description of the genitalia.

Two specimens were dissected. Penis cylindrical, relatively long; retractor muscle short, attaches on the proximal portion; penial caecum very long, with a thickened base and a cylindrical distal portion; an additional canal (?) connects the proximal end of the epiphallus with the penial caecum; epiphallus long, with a thickened distal part; the separation between the vas deferens and epiphallus is distinct; vas deferens relatively thick. Vagina cylindrical and relatively short; bursa copulatrix extremely long with a pointed end.

A developing egg covered with small calcareous crystals was found in the uterus of the figured specimen. In the other specimen, an elongated, simple bursa copulatrix was found with a slightly thickened apical part.

##### Distribution.

South-Eastern Bulgaria and North-Western Turkey.

##### Ecology.

The type series (12 shells) of *Orcula zilchi* was collected by Urbański on the floodplain of the Ropotamo River in leaf litter and under decaying wood. It was found in association with *Sphyradium doliolum* (Bruguière 1792), *Oxychilus deilus rumelicus* Hesse, *Laciniaria plicata* (Draparnaud), *Bulgarica denticulata thessalonica* (Rossmässler), *Euxina persica paulhessei* (Lindholm), *Euxina circumdata* (L. Pfeiffer), *Cochlodina laminata* (Montagu). Atanas Irikov visited the type locality (very humid forest with rocks along the river) on two occasions, with collection time totalling 6–8 hours. Besides *Orcula zilchi* he collected all other species previously reported from the Ropotamo area.

We were able to find *Orcula zilchi* only in deciduous forests. In Bulgaria (near Kondolovo village), living specimens were collected in an oriental beech (*Fagus orientalis*) forest in shady and moist microhabitats between the leaf litter and soil. These conditions were very similar to the Abant Gölü locality (Turkey). The other Turkish locality (between Bozüyük and İnegöl) was slightly different, with a deciduous forest at the base of a large limestone rock, on a slope covered with smaller stones and larger rocks.

The species is very rare wherever it has been encountered yet, especially in Turkey. On two occasions, in 2007 and 2010, Barna Páll-Gergely spent about 4–5 hours at the locality in vil. Bursa, but found only one specimen in 2007. The other locality (Vil. Bolu) was visited in 2005 and 2006 for similar lengths of time and only one specimen was found in 2006. Atanas Irikov collected 9 living specimens and about 10 empty shells in an hour near Kondolovo in Bulgaria.

##### Conservation status.

Listed as Vulnerable (V) under IUCN criteria (Pall-Gergely 2011b). Deforestation and disturbance of the forests are the main threat to this species.

##### Remarks.

Two of four living specimens had beetle (possibly drilid beetle) larvae in the body whorl.

The dissected specimens were collected about 23 km south-southwest of the type locality. The Strandzha Mountains (incl. the collecting site) belongs to the drainage of the Ropotamo River. It is reasonable to suppose that Urbański’s population was “washed down” from somewhere in the Strandzha Mts. and settled a temporary subpopulation in the Ropotamo floodplain. This might be a reasonable explanation why A. Irikov could not find this species in the type locality.

#### Genus Sphyradium

Charpentier 1837

http://species-id.net/wiki/Sphyradium

Sphyradium doliolum (Bruguière 1792)
Bulimus doliolum Bruguière Encyclopédie méthodique: 351. (1792)
Sphyradium dobrogicum Grossu, **new synonym** Travaux du Muséum d’Histoire Naturelle ‘’Grigore Antipa’’ 28: 7–13. Bucureşti. [Dobrogea, département de Tulcea, près du Monastère Cocoş de la Forêt Luncaviţa.] (1986)
Sphyradium dobrogicum , — Grossu: Gastropoda Romaniae 2: 228–230, fig. 120. [‘’pădurea Luncaviţa în apropierea Mănăstirei Cocoş, jud. Tulcea’’]. (1987)
Orcula dobrogica , — Welter-Schultes, European non-marine molluscs:143. (2012)

##### Remarks.

*Sphyradium dobrogicum* was described based on a single shell. The holotype could not be located in the collection of the Grigore Antipa National Museum of Natural History (Bucharest) during a recent search (2012). It could still be in Grossu’s house (Oana Popa, pers. comm., 2011) but, at present, the holotype seems to be lost.

Bank (2011) and Welter-Schultes (2012) assigned the species to *Orcula* without supporting evidence. *Sphyradium dobrogicum* has a domed apex, a ribbed shell and very weak lamellae (see original description and drawing), indicating that it may represent a dwarf specimen of *Sphyradium doliolum*. We visited the type locality of *Sphyradium dobrogicum* in 2011 but found only *Sphyradium doliolum*.

Based on available information we suggest using *Sphyradium dobrogicum* as a synonym of *Sphyradium doliolum*.

### *Orcula gularis* and *Orcula dolium* in Romania

***Orcula gularis***: Bielz (1863, 1867) reported *Pupa (Pupilla) gularis* from Gușterița, which is presently part of Sibiu. Grossu (1987, 1993) cited this record in his account of *Sphyradium gularis*. Grossu (1986) discussed Bielz’s specimens housed in the museum in Sibiu, but specimens were not located in the collection of Bielz in the NHMS. Although Bank (2011) reports the species from Romania, recent authors consider this record as erroneous (Falkner et al. 2011) or simply ignore it (Welter-Schultes 2012). Indeed, the occurrence of *Orcula gularis* in Romania, more than 650 km from its main distribution area is very unlikely. However, to our knowledge, no one has searched for the species at the respective Romanian locality which was well-defined by Bielz. Nevertheless, despite intensive collecting efforts by competent malacologists over the last 150 years, *Orcula gularis* has not been encountered in Transylvania.

*Orcula gularis* is a very characteristic species with a strong palatal tooth in the aperture. Today, with literature available it is very difficult to misidentify *Orcula jetschini* as *Orcula gularis* Based on recent available literature, these two species easily can be distinguished from each other. Bielz also had non-Romanian comparative material of *Orcula gularis* at his disposal: Krain (NHMSB 139634–139635), Oberkrain (NHMSB 139636–139639), Karnthen (NHMSB 139640–139641), Hohewand (NHMSB 139642–139644) and Austria (NHMSB 139645–139646) (Ana Mesaroş, pers. comm.). On the other hand, the understanding of the Orculidae was insufficient at that time. For example, Bielz confused *Orcula jetschini* with *dolium* (see below). Perhaps, a thickening behind the aperture lip of the examined specimens led Bielz to misidentify them as *Orcula gularis* instead of *Orcula jetschini*, the species inhabiting that area.

***Orcula dolium***: *Orcula dolium* has been recorded from Romania by several authors. Bielz (1863) reported it from ‘’Vajda-Hunyad am Kaczanyas’’ (Hunedoara), ‘’Valea-Ordinkusi bei Skerisora’’ (Ordâncuşa valley at Scărișoara), ‘’nördlich von Unter-Grohot bei Körösbánya’’ (Baia de Criș), ‘’Vormága’’ (Vărmaga) and ‘’Collegiumwald von Nagy-Enyed’’ (Aiud). In the collection of Bielz (NHMS) we only found the sample from Körösbánya, one sample from ‘’Hideg-Szamos’’ and more samples from Klausenburg (Cluj Napoca). Kertész (1901) reported *Orcula dolium* from a few localities in the Bihor Mountains, and Grossu (1987) most recently from a number of localities, namely from northern Oltenia and the Banat area, and a remote locality in Tulcea County (Luncaviţa, around the Cocoş Monastery).

Soós (1943) speculated that records of *Orcula dolium* by Bielz and Kertész (1901) from the Apuseni Mountains should be assigned to *Orcula jetschini*. In fact, Bielz revised his original labels from *Pupa dolium* to *jetschini* (NHMS).

Two Romanian samples of Grossu in the collection of MNINGA are labelled as *Orcula dolium*: MNINGA GST/923, Horezu, Valcea, leg. Grossu (3 shells) and MNINGA 28142, Bucegi, ‘’Sertarul 114’’, ex Licherdopol (‘’*Orcula dolium* var. *implicata*’’, 3 shells). Both samples are actually *Sphyradium doliolum*. We cannot explain the Luncaviţa locality (see remarks under *Sphyradium dobrogicum*). We collected near the localities mentioned by Grossu (Oltenia and Banat) and found only *Orcula jetschini*. Mitrović (2007) reported *Orcula dolium* from the Pleistocene of the Serbian Kostolac, very close to Grossu’s localities. It is possible that some of the specimens Grossu examined are Pleistocene fossils, but it seems unlikely that *Orcula dolium* still lives in the Banat-Oltenia area in Romania. Soós (1943) marked Torna (southeast Slovakia) as the easternmost locality of *Orcula dolium*.

## Discussion

In this paper we describe the genitalia of the Eastern European *Orcula jetschini* and *Orcula zilchi* for the first time. We also examine and describe the anatomy of *Orcula wagneri* from a locality that lies 200 km north of populations examined by Schileyko (2012). This additional information and data published by other authors (mainly Gittenberger 1983 and Schileyko 2012) allows us to review the taxonomic relationships of the entire genus.

The genus can be subdivided into three subgenera (*Orcula*, *Illyriobanatica* subgen. n. and *Hausdorfia* subgen. n.) based on the shell characters and the morphology and size of the penial caecum, which serves as the primary diagnostic character. Unpublished results of molecular phylogenetic analysis (Harl et al. in prep.) of most *Orcula* species and subspecies indicate the monophyly of these three groups. This subdivision is in good agreement with biogeographic information.

Three species included herein have some shell and anatomical characters which differ from characters used to the features mentioned in the diagnoses of certain subgenera: (1) the shell sculpture of many populations of *Orcula (Illyriobanatica) wagneri* is almost smooth, which is unusual in the subgenus. (2) The penial caecum of *Orcula (Orcula) restituta* is very short compared to other species assigned to the subgenus. (3) The penial caecum of *Orcula (Illyriobanatica) schmidtii transversalis* is short, but simple (Hausdorf 1987), not ‘’tuberculated’’ as the other forms of the subgenus. The caecum of *Orcula schmidtii transversalis* seems to be double in the illustration of Reischütz and Sattmann (1990).

Based on available literature, the occurrence of *Orcula (Orcula) dolium* and *Orcula (Orcula) gularis* in Romania is discussed. Most purported Romanian ‘’voucher specimens’’ are lost or we were unable to examine them. As the published literature is based on possibly misidentified specimens, and the verified distributional ranges of *Orcula gularis* and *Orcula dolium* lie far from the Romanian records, we suggest deleting *Orcula gularis* and *Orcula dolium* from the Romanian faunal list. The occurrence of only one *Orcula* species, namely *Orcula (Illyriobanatica) jetschini* is verified from Romania.

## Supplementary Material

XML Treatment for Orcula

XML Treatment for Orcula

XML Treatment for Orcula (Illyriobanatica)

XML Treatment for Orcula (Illyriobanatica) jetschini

XML Treatment for Orcula (Illyriobanatica) wagneri

XML Treatment for Orcula (Hausdorfia)

XML Treatment for Orcula (Hausdorfia) zilchi

XML Treatment for Sphyradium
